# Psychiatrische Versorgung in einer muttersprachlichen Ukraine-Ambulanz für Geflüchtete in einer psychiatrischen Universitätsklinik in Deutschland

**DOI:** 10.1007/s00115-024-01661-1

**Published:** 2024-04-19

**Authors:** Daniel Kamp, Timo Jendrik Faustmann, Nadiya Kovach, Jana Lüdtke, Maria Luisa Schiffers, Michaela Jänner, Leonhard Schilbach

**Affiliations:** 1 Klinik und Poliklinik für Psychiatrie und Psychotherapie, Medizinische Fakultät, Heinrich-Heine-Universität Düsseldorf, Universitätsstraße 1, 40225 Düsseldorf, Deutschland; 2 Abteilung für Allgemeine Psychiatrie 2, LVR-Klinikum Düsseldorf, Bergische Landstraße 2, 40629 Düsseldorf, Deutschland; 3 Klinik für Psychiatrie und Psychotherapie, Ludwig-Maximilians-Universität München, Nußbaumstraße 7, 80336 München, Deutschland

## Einleitung

Seit Beginn des Angriffskriegs Russlands auf die Ukraine am 24. Februar 2022 sind über eine Million Menschen aus der Ukraine nach Deutschland geflüchtet [10].

Hieraus resultiert ein sektorübergreifender Bedarf für das deutsche Gesundheitssystem, der sich im Fall von psychiatrischen Erkrankungen aus zwei verschiedenen Patientenpopulationen herleiten lässt:

Einerseits besteht die Notwendigkeit zur Weiterbehandlung psychiatrisch vorerkrankter Flüchtlinge. Andererseits stellen Kriegs- und Fluchterlebnisse Belastungsfaktoren und Traumata dar, die zu Anpassungsstörungen und Traumafolgestörungen führen können oder zur Erstmanifestation anderer psychiatrischer Erkrankungen beitragen. Kriegsflüchtlinge leiden oft (in absteigender Reihenfolge) unter Depression und posttraumatischer Belastungsstörung (PTBS), Angststörung und Psychose [1] und benötigen häufig über viele Jahre hinweg psychiatrische Hilfen [6].

Somit besteht ein hoher Bedarf an qualifizierter psychiatrisch-psychotherapeutischer Hilfe, die ambulante Regelversorgung wird jedoch häufig durch Sprachbarrieren erschwert [10].

Deshalb wurde seit Kriegsbeginn eine an unsere psychiatrische Institutsambulanz angeschlossene muttersprachliche Ambulanz für aus der Ukraine Geflüchtete („Ukraine-Ambulanz“) etabliert.

Ziel dieser retrospektiven Auswertung der Patient*innendaten dieser Ukraine-Ambulanz ist die Charakterisierung der Patient*innenbedarfe zur Darstellung etwaiger Unterschiede und weiteren bedarfsgerechten Optimierung der medizinischen Versorgung.

## Methode

Es erfolgte an einer deutschen psychiatrischen Universitätsklinik eine Vollerhebung aller ambulanten Fälle, die im Beobachtungszeitraum (01.04.2022–31.03.2023) die sogenannte Ukraine-Ambulanz erstmalig konsultiert haben.

In einem ersten Schritt wurden die elektronisch im Krankenhausinformationssystem vorliegenden soziodemografischen Daten dieser Fälle mit allen anderen Erstkontakten unserer Institutsambulanz im Beobachtungszeitraum verglichen.

In einem zweiten Schritt wurde computerbasiert jedem Patienten der Ukraine-Ambulanz ein im Hinblick auf Alter und Geschlecht gematchter Kontrollpatient aus der Institutsambulanz zugeordnet. Für diesen Auswertungsschritt wurden die elektronisch verfügbaren Daten des Schritts eins durch eine manuelle Suche in den Patientenakten ergänzt.

Der Vergleich nominalverteilter Daten erfolgte mithilfe des exakten Fisher-Freeman-Halton-Tests, der Vergleich mindestens intervallskalierter Daten mit T‑Tests. Es wurde von einem Signifikanzlevel bei *p* < 0,05 (zweiseitig) ausgegangen.

Vor Studienbeginn wurde durch die Ethikkommission der Heinrich-Heine-Universität Düsseldorf ein positives Ethikvotum (Studien-Nr.: 2023-2420) ausgesprochen. Die Studie steht im Einklang mit nationalem Recht sowie gemäß der Deklaration von Helsinki von 1975 (in der aktuellen, überarbeiteten Fassung).

## Ergebnisse

Im Beobachtungszeitraum konsultierten 67 Patient*innen erstmalig die Ukraine-Ambulanz bei 432 erstmaligen Konsultationen der allgemeinen Institutsambulanz. Das mittlere Alter bei Erstkonsultation der Ukraine-Ambulanz unterschied sich mit 36,42 ± 12,32 Jahren (Mittelwert ± Standardabweichung) nicht signifikant vom Alter bei Erstkonsultation der Institutsambulanz, das bei 35,67 ± 12,36 Jahren lag (Altersbereich 18–65 Jahre). Hinsichtlich der Geschlechtsverteilung wurde die Ukraine-Ambulanz hochsignifikant häufiger (*p* < 0,001) von Frauen (57 von 67 = 85 %) konsultiert als die Institutsambulanz (208 von 432 = 48 %; *p* < 0,001).

Im Vergleich mit der im Hinblick auf Alter und Geschlecht gematchten Kontrollgruppe zeigte sich für die Patient*innen der Ukraine-Ambulanz eine signifikant höhere ambulante Konsultationsfrequenz bei signifikant geringerer stationärer Aufnahmehäufigkeit in unserer Klinik. Hierzu ist anzumerken, dass die Patient*innen signifikant häufiger nicht aus Düsseldorf kamen. Die Patient*innen der Ukraine-Ambulanz waren signifikant seltener berufstätig. Hinsichtlich Familienstand, durchschnittlicher Anzahl der Kinder, Schulbildung und psychiatrischer Vorbehandlung unterschieden sich die beiden Gruppen nicht signifikant. Tab. 1 fasst die soziodemografischen Daten zusammen.

**Tab. 1:** Soziodemografische Daten der Ukraine-Ambulanz sowie der allgemeinpsychiatrischen Ambulanz

	Ukraine-Ambulanz	Gematchte Kontrollgruppe	*p*-wert
*Anzahl ambulanter Kontakte (MW* *±* *SD)*	4,45 ± 3,58	2,99 ± 3,93	0,016
*Anzahl stationärer Aufnahmen (MW* *±* *SD)*	0,13 (± 0,55)	0,67 ± 1,01	< 0,001
*Anzahl Kinder (MW* *±* *SD)*	1,13 (± 1,07)	1,07 (± 1,20)	0,787
*Wohnort Düsseldorf*	29/67	47/67	0,003
**Familienstand:**			0,628
*Nicht in einer Beziehung*	13	12	–
*In einer festen Beziehung*	8	10	
*Verheiratet*	14	9	
*Geschieden*	10	6	
*Verwitwet*	4	1	
**Schulabschluss:**			0,892
*Schüler*	0	0	–
*Ohne Hauptschulabschluss*	0	0	
*Hauptschul‑/Realschulabschluss *(entspricht grundlegender Sekundarschulbildung in der Ukraine)	4	1	
*Fachhochschulreife *(ukrainischer Patient schon länger in Deutschland und Schulabschluss erworben)	1	1	
*Abitur *(entspricht vollständiger Sekundarschulbildung in der Ukraine)	21	15	
**Aktuell berufstätig:**			< 0,001
*Ja*	9	23	–
*Nein*	39	15	
**Psychiatrische Vorbehandlung:**			0,202
*Ja*	42	47	–
*Nein*	18	13	

**Abb. 1:**
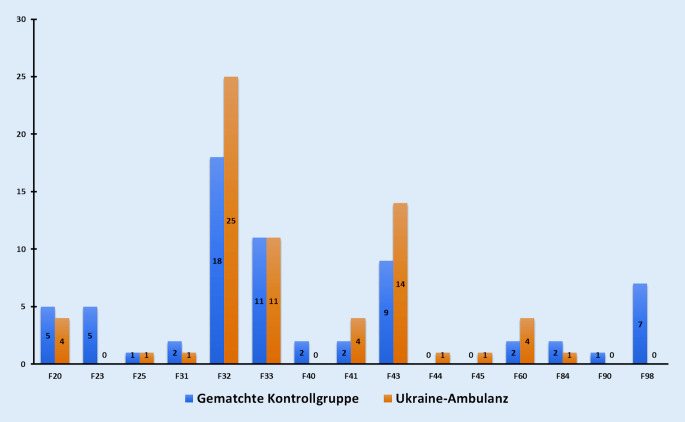
Die Diagnoseverteilung (mittels Chi^2^-Test) ergab einen signifikanten Unterschied zwischen der allgemeinpsychiatrischen Ambulanz (*blau*) und der Ukraine-Ambulanz (*orange; x‑Achse*). Anzahl der Patienten (*y‑Achse*)

Bezüglich der Diagnoseverteilung unterschied sich die Patientengruppe der Ukraine-Ambulanz signifikant von der gematchten Kontrollgruppe (Chi^2^: 21,365; *p* = 0,039), wobei sich deutliche Unterschiede im Bereich der Reaktionen auf schwere Belastungen und Anpassungsstörungen (ICD-10: F43; Ukraine-Ambulanz: 14; Institutsambulanz: 9) sowie depressiven Episoden (ICD-10: F32; Ukraine-Ambulanz: 25; Institutsambulanz: 18) zeigten. Abb. 1 fasst die Diagnoseverteilung beider Gruppen zusammen. Die Diagnose einer PTBS wurde in der Ukraine-Ambulanz dreimal, in der Institutsambulanz einmal vergeben.

## Diskussion

Unsere Ukraine-Ambulanz wurde, im Einklang mit vergleichbaren Studien, bei denen der Frauenanteil bei 79,8 % bzw. 85,5 % lag [8, 9], signifikant häufiger von Frauen genutzt, möglicherweise aufgrund der Ausreiseerschwernisse für ukrainische Männer.

Auffällig ist, dass sich Ukraine-Ambulanz und Kontrollgruppe im Hinblick auf die psychiatrische Vorbehandlung nicht unterscheiden. Unsere Ukraine-Ambulanz wird primär von bereits vor der Flucht psychiatrisch erkrankten Patient*innen konsultiert und weniger durch vor der Flucht Gesunde. Möglicherweise besteht, trotz Muttersprachlichkeit des Angebots, eine Stigmatisierungsangst bei vor der Flucht gesunden Ukrainerinnen und Ukrainern. Die Problematik, dass aus der Ukraine Geflüchtete tendenziell weniger psychiatrische Hilfe suchen, ist vorbeschrieben. Hierzu zeigten unter einer gezielten Befragung von 1347 ukrainischen Kriegsflüchtlingen in Tschechien 41 % eine moderate oder schwere depressive Symptomatik und 23 % eine moderate oder schwere Angststörung, jedoch war der Anteil derer, die diesbezüglich Hilfe gesucht haben, niedrig [5]. Dies könnte den niedrigen Anteil derer, die in unserer Kohorte erstmalig eine psychiatrische Symptomatik entwickelt haben, erklären. Der Wohnort der Patient*innen, die die Ukraine-Ambulanz aufsuchten, unterschied sich signifikant von der Kontrollgruppe, bei der schwerpunktmäßig Patient*innen aus dem Düsseldorfer Einzugsgebiet eine psychiatrische Konsultation suchten. Hierbei ist anzumerken, dass die Patient*innen der Ukraine-Ambulanz teilweise aus ganz Nordrhein-Westfalen kamen, was als Hinweis für ein fehlendes flächendeckendes Angebot für diese Patientengruppe interpretiert werden kann.

### Bereits vor der Flucht erkrankte Patient*innen suchten die Ukraine-Ambulanz auf

Ukrainischsprachige Patient*innen waren in unserer Kohorte trotz vergleichbarer Schulbildung signifikant seltener berufstätig, wahrscheinlich, da nach der Flucht noch keine Etablierung einer Erwerbstätigkeit erfolgen konnte. Hinsichtlich der Anzahl der Kinder und Schulbildung unterschieden sich beide Kohorten weder untereinander noch von Angaben in der Literatur signifikant, wobei unsere Kohorte mit einem Durchschnittsalter von 36,4 Jahren etwa zehn Jahre jünger und seltener verheiratet war als in Vergleichsarbeiten, bei denen Geflüchtete in Polen oder der Ukraine selber (also innerhalb des Kriegslands umgesiedelte Leute) sowie über Europa verteilte Geflüchtete untersucht wurden. Hier wurde jedoch im Vergleich zu unserer Kohorte (Altersbereich 18–65 Jahre) keine weitere Angabe zur Altersgrenze erwähnt [8, 9].

Das Diagnosespektrum der Ukraine-Ambulanz unterschied sich signifikant von der Kontrollgruppe mit einem Überwiegen von affektiven Erkrankungen und Belastungsstörungen, was aufgrund der Belastungsfaktoren eines Kriegsgeschehens wenig überrascht.

Auffällig ist, dass in unserer Kohorte trotzdem nur dreimal (= 4,5 %) eine PTBS diagnostiziert wurde, wobei eine Diagnoseprävalenz von PTBS bei Kriegsflüchtlingen allgemein von ca. 31 % in der Literatur diskutiert wird [1]. Möglicherweise ist in unserer Patientenkohorte der Anteil an Patient*innen, die einem traumatischen Ereignis ausgesetzt waren, im Vergleich zu anderen Kohorten von Kriegsflüchtlingen geringer. So ist bekannt, dass z. B. die direkte Beteiligung an bewaffneten Konflikten ein relevanter Risikofaktor für die Entstehung einer PTBS ist [7]. In unserer Kohorte konnten wir keine weiteren Informationen bezüglich einer möglichen direkten Beteiligung an bewaffneten Auseinandersetzungen erhalten.

Interessanterweise wurde zuletzt berichtet, dass eine Unterstützung durch soziale Netzwerke, Gastfamilien sowie die Fortsetzung der Lebensgewohnheiten einen Resilienzmechanismus darstellen [2, 8]. Weiter wurde bereits diskutiert, dass im Rahmen des Ukrainekonflikts unter nach Polen Geflüchteten der Anteil an psychischen Erkrankungen unter Frauen höher ist sowie unter Geflüchteten, die die Sprache im Ankunftsland nicht sprechen, und dass psychologischer Stress hier eher bei jüngeren Leuten auftritt [4]. Vergleichbare Daten aus Deutschland ergaben nach einer Fragebogenerhebung ebenfalls einen signifikant höheren Anteil von psychologischem Stress, depressiven Symptomen und Angst unter weiblichen Geflüchteten aus der Ukraine und diese Angaben waren weiter mit einer schlechteren Lebensqualität assoziiert [3].

## Fazit für die Praxis

Auf Grundlage dieser Ergebnisse sollte die ambulant-psychiatrische Versorgung ukrainischsprachiger Geflüchteter in Deutschland Angebote mit einem Schwerpunkt für Frauen im Bereich affektiver Erkrankungen oder Traumafolgestörungen vorhalten.
